# Role for the ATPase inhibitory factor 1 in the environmental carcinogen-induced Warburg phenotype

**DOI:** 10.1038/s41598-017-00269-7

**Published:** 2017-03-15

**Authors:** Kévin Hardonnière, Morgane Fernier, Isabelle Gallais, Baharia Mograbi, Normand Podechard, Eric Le Ferrec, Nathalie Grova, Brice Appenzeller, Agnès Burel, Martine Chevanne, Odile Sergent, Laurence Huc, Sylvie Bortoli, Dominique Lagadic-Gossmann

**Affiliations:** 1 Inserm U1085, Institut de Recherche en Santé, Environnement, Travail, Rennes France; 2 Université de Rennes 1, Biosit UMS3080, 35043 Rennes Cédex, France; 3 Institute of Research on Cancer and Ageing of Nice (IRCAN), INSERM U1081, CNRS UMR7284, Université de Nice-Sophia Antipolis, Faculté de Médecine, Centre Antoine Lacassagne, Nice, F-06107 France; 4 HBRU, Luxembourg Institute of Health, 29, rue Henri Koch, L-4354 Esch-sur-Alzette Luxembourg; 5 INRA UMR 1331 ToxAlim, Toulouse, France; 6 INSERM UMR-S 1124, Université Paris Descartes, Centre Universitaire des Saint-Pères, Paris, France

## Abstract

Most tumors undergo metabolic reprogramming towards glycolysis, the so-called Warburg effect, to support growth and survival. Overexpression of IF1, the physiological inhibitor of the F0F1ATPase, has been related to this phenomenon and appears to be a relevant marker in cancer. Environmental contributions to cancer development are now widely accepted but little is known about the underlying intracellular mechanisms. Among the environmental pollutants humans are commonly exposed to, benzo[a]pyrene (B[a]P), the prototype molecule of polycyclic aromatic hydrocarbons (PAHs), is a well-known human carcinogen. Besides apoptotic signals, B[a]P can also induce survival signals in liver cells, both likely involved in cancer promotion. Our previous works showed that B[a]P elicited a Warburg-like effect, thus favoring cell survival. The present study aimed at further elucidating the molecular mechanisms involved in the B[a]P-induced metabolic reprogramming, by testing the possible involvement of IF1. We presently demonstrate, both *in vitro* and *in vivo*, that PAHs, especially B[a]P, strongly increase IF1 expression. Such an increase, which might rely on β2-adrenergic receptor activation, notably participates to the B[a]P-induced glycolytic shift and cell survival in liver cells. By identifying IF1 as a target of PAHs, this study provides new insights about how environmental factors may contribute to related carcinogenesis.

## Introduction

The mitochondrial H^+^-ATP synthase, also called F0F1ATPase or complex V, is a master regulator of energy production and cell fate^1–3^. Indeed, besides its well-recognized physiological role in oxidative phosphorylation (OXPHOS) as the major cell producer of ATP, this enzyme has been implicated in the morphogenesis of mitochondrial cristae^4^, in the formation of the mitochondrial permeability transition pore (mPTP) during cell death^5^, and the metabolic reprogramming of tumor cells^6^. Regarding this latter point, a decreased OXPHOS capacity and subsequent drop in ATP synthesis due to complex V inhibition, appears to be responsible for a metabolic shift towards aerobic glycolysis, which is better known as the Warburg effect^1^. In this context, OXPHOS inhibition is often linked to an apoptotic-resistant phenotype, and complex V regulation thus appears to be essential for tumor progression^1, 6^.

Among the known regulators of the H^+^-ATP synthase, the physiological inhibitor ATP Inhibitory Factor 1 (IF1) has been implicated in the short-term regulation of energy metabolism by directly interacting with the βF1 subunit of this pump, whereby inhibiting its ATP hydrolysis activity^7^. IF1 protein, in its native form, is present as a tetramer in mitochondrial matrix at a physiological matrix pH of ∼8.0. When matrix acidifies, a release of the active dimeric form of IF1 occurs, triggering the IF1 binding to the β-F1 subunit, thus preventing complex V reverse activity^8^. Indeed, complex V activity is able to switch from ATP synthase activity to ATP hydrolase activity under certain circumstances in order to sustain mitochondrial membrane potential (Δψm), and hence mitochondria integrity; however, this could inexorably sentence cell to death due to ATP hydrolysis if no complex V inhibition occurs^9, 10^. Interestingly, an increased IF1 level has been involved in the establishment of a high Δψm and the conservation of ATP^11, 12^. Besides, Sánchez-Aragó and coworkers have evidenced high IF1 levels in diverse human cancers, thus highlighting its relevance as a predictive marker for clinical outcome^13^. In line with this, a recent paper described an increased expression of IF1 in human hepatocellular carcinoma (HCC), such a high increase being predictive of poor survival^14^. Regarding IF1 roles in tumorigenesis, its overexpression has been involved in the acquisition by cells of several cancer phenotype hallmarks, including metabolic reprogramming^11, 14, 15^, increased proliferation and invasion^14^, and cell evasion from death^16, 17^. Angiogenesis would also be targeted by IF1^14^. Altogether these observations emphasize the key role IF1 might play in tumor development. However, the precise mechanisms underlying the IF1 increase during tumorigenesis remain poorly described.

One clue might come from exposure to environmental carcinogens such as polycyclic aromatic hydrocarbons (PAHs). These widespread contaminants are notably found in cigarette smoke, exhaust fumes, grilled meat, among others, and have been related to tumor development, notably in lung and liver^18, 19^. We recently demonstrated that a low concentration of benzo[a]pyrene (B[a]P), the prototype molecule of PAHs which is classified as human carcinogen of group 1 by IARC^20^, not only hyperpolarized mitochondria^21^, but also induced both a mitochondrial matrix acidification, a glycolytic shift, and an EMT/migration phenotype of liver cells^22^. Furthermore, a reverse activity of complex V upon B[a]P exposure, was also previously suggested^23^. As all these cell responses have been linked to IF1, the present study therefore aimed at testing the impact of B[a]P on IF1 level, and at evaluating its role in the survival process elicited by this contaminant in the F258 rat liver epithelial cell line. Here we show that PAHs can increase *in vivo* the IF1 content in rat liver. We further demonstrate that the IF1 up-regulation observed *in vitro* upon B[a]P exposure would rely on the activation of the β2 adrenergic receptor pathway, and that it would be determinant in both the glycolytic shift and cell survival elicited by this compound.

## Methods

### Chemicals

Benzo[a]pyrene (B[a]P), α-naphthoflavone (α-NF), propranolol, ICI-118,551 and 1-methyl – N - [2 – methyl – 4 -[2 - (2 - methylphenyl) diazenyl] phenyl - 1H – pyrazole – 5 -carboxamide (CH223191), were all purchased from Sigma Chemical Co. (St. Louis, MO). Hoechst 33342 and MitoTracker Red CMXROS were purchased from Life Technologies (Saint-Aubin, France). All these products were used as a stock solution in DMSO; final concentration of this vehicle in culture medium was <0.00005% (v/v), and control cultures received the same concentration of vehicle as treated cultures.

Monoclonal mouse anti-ATP synthase subunit beta (A-21351) antibody was purchased from Life Technologies (Saint-Aubin, France). Polyclonal rabbit anti-ATPIF1 antibody (#8528; Cell Signalling) and monoclonal rabbit anti-COX IV antibody (#4850; Cell Signalling) were purchased from Ozyme (Montigny-le-Bretonneux, France). Monoclonal mouse anti-HSC70 antibody (sc-7298) was purchased from Santa Cruz Biotechnology (Heidelberg, Germany). Polyclonal rabbit anti-AhR antibody (BML-SA550) was purchased from Enzo Life Sciences (Lyon, France). Secondary antibodies conjugated with horseradish peroxidase were purchased from DAKO (Les Ulis, France).

The sixteen PAHs (naphthalene, fluorene, acenaphthene, acenaphthylene, anthracene, phenanthrene, fluoranthene, pyrene, benzo[a]anthracene, chrysene, benzo[b]fluoranthene, benzo[k]fluoranthene, benzo[a]pyrene, benzo[g,h,i]perylene, indeno[1,2,3-cd]pyrene and dibenzo[a,h]anthracene) used for the animal experiment model were purchased from Sigma Aldrich (Bornem, Belgium).

### Gene expression microarray and Gene Set Enrichment Analyses (GSEA)

We used GSEA^24^ to identify pathways and gene sets associated with variation in ATPIF1 mRNA expression levels across 91 hepatocellular carcinomas^25^ (GSE20238). Genes were sorted by their concordance (Pearson correlation) with ATPIF1 mRNA expression levels across tumors, and GSEA was used to evaluate gene sets enriched for either negatively or positively correlated genes. Published GSE20238 was downloaded from the InSilico DB Genomic Datasets Hub^26^ (https://insilicodb.com/), and analyzed by using the GSEA v2.07 software (http://www.broad.mit.edu/gsea), as previously described^24^. To account for gene-gene correlations in the enrichment analysis, GSEA p values were computed with respect to a null distribution obtained from 1,000 randomizations of the patient-phenotype labels. Enriched gene sets were selected on the basis of statistical significance (false discovery rate FDR q value < 0.25, and normalized p value < 0.05). Heatmap visualization was performed using GENE-E (http://www.broadinstitute.org/).

### Animal Experimentation

#### Animal housing

Fifteen Long Evans rats (female of 180–200 g, Elevage Janvier, St Berthevin, France) were housed in plastic cages under controlled environment (12 h light/dark cycle, light on at 7 am, temperature of 22 ± 2 °C and relative humidity of 40 ± 5%). Food and water were available *ad libitum*. The water, food and oil were tested according to NF ISO 15302 to confirm that all these matrices were PAH-free down to a detection limit of 10 ng/L of water and 1 ng/g of fat. Rats were acclimatized to the animal facility for 2 weeks prior to experiment onset.

#### Animal treatment

The mix of PAHs was composed of the 16 compounds pointed out by the US-Environmental Protection Agency (US-EPA) for their toxicity, and prepared in vegetable oil weekly (ISIO4, Lesieur, Neuilly-sur-Seine, France). Five rats were randomly allocated to each of the experimental groups receiving 0.04 and 0.8 mg/kg body weight of each compound included in the mix, by oral administration, 3 times per week over a 90-day period. The exposure levels were far below the LD50 for all the molecules tested and determined on the basis of a previous study^27^. Control rats received only the vehicle. At the end of the 90 days-experiment, the rats were euthanized 3 hours after the last gavage by using carbon dioxide. A cardiac puncture was performed after the 3 minutes of unconsciousness and just before the heart stopped beating. All procedures were conducted in accordance with European Communities Council Directive of 22 September 2010 (2010/63/EU) and approved by the Ministry of Agriculture, Grand-Duchy of Luxembourg.

#### Tissue collections

Livers were dissected and weighed. The base of the left lateral liver lobe was divided into 5 equivalent pieces of 100 mg each which were placed in cryogenic tubes, frozen in liquid nitrogen and stored at −80 °C until analysis.

#### Liver sample preparation

These 100 mg of tissue were lysed on ice, using a potter, in 600 µl of RIPA buffer supplemented with 1 mM phenylmethylsulfonyl fluoride, 0.5 mM dithiothreitol, 1 mM orthovanadate, and a cocktail of protein inhibitors (Roche, Meylan, France), and were sonicated for 10 seconds. Lysates were then centrifuged at 14,000 g for 15 min at 4 °C. The resulting supernatants were collected and frozen at −80 °C or used immediately. Samples were finally diluted 1/50 before protein quantification for western blotting.

### Cell culture

The F258 rat liver epithelial cell line was cultured in Williams’ E medium supplemented with 10% fetal calf serum, 2 mM L-glutamine, at 37 °C under a 5% CO_2_ atmosphere and treated 24 h following seeding as previously described^21, 28^. F258 cells were treated with B[a]P for 24, 48 and 72 h. Cells were seeded at a density of 2.5× 10^3^ cells/cm^2^ one day before treatment, and the medium changed before exposures. When using inhibitors, these molecules were added 1 h before B[a]P treatment, for the indicated time points.

### RNA isolation and reverse transcription – real-time quantitative polymerase chain reaction (RT-qPCR) analysis

Total RNAs were isolated from F258 cells using the TRIzol method (InVitrogen) and were then subjected to RT-qPCR analysis, as previously described^29^. Gene-specific primers for ATPIF1 and 18S were purchased from Sigma, and the sequences used for each gene were as follows: ATPIF1: forward, ACGCCGAAGATAATGGCAGG – reverse, ATCCATGCTCTCCGACGAGT; 18S: forward, CCGGTACAGTGAAACTGCGA – reverse, GATAAATGCACGCGTTCCCC. The amplification curves of the PCR products were analyzed with the ABI Prism SDS software using the comparative cycle threshold method. To assess the successful amplification of each target gene, a standard curve was performed for each primer set. The expression levels of target genes were normalized relative to the expression of an 18S RNA endogenous reference and were plotted as fold change compared to control with vehicle (DMSO).

### Mitochondrial purification

Briefly, after treatments cells were washed twice with cold PBS. Then, cells of each 150 mm petri dishes were directly scratched in 1.5 ml of cold hypotonic buffer (20 mM HEPES pH 7.5, 10 mM KCl, 1.5 mM MgCl_2_, 1 mM EDTA, 250 mM sucrose) supplemented with 100 µM PMSF and complete protease inhibitor cocktails (Roche, Meylan, France). Lysates were transferred into 1.5 ml tubes before starting lysis with a 26G needle syringe. Samples were kept under stirring at 4 °C for 1 hour to improve lysis efficiency. A first 10 min, 750 g centrifugation was performed at 4 °C. Supernatants were collected and a second 25 min, 10,000 g centrifugation was performed at 4 °C. Mitochondria pellets were then lysed in RIPA buffer supplemented with 1 mM phenylmethylsulfonyl fluoride, 0.5 mM dithiothreitol, 1 mM orthovanadate, and a cocktail of protein inhibitors (Roche). Mitochondrial fractions were finally centrifuged at 13,000 g for 15 min at 4 °C. The resulting supernatants were collected and frozen at −80 °C or used immediately.

### Western Blot immunoassays

See Supplementary Information.

### Intracellular Calcium Measurements

F258 cells were cultured on glass coverslips and incubated with 2.5 μM Fura-2-AM for 20 min at 37 °C in Hepes-buffered medium (10 mM Hepes, 134.8 mM NaCl, 4.7 mM KCl, 1 mM MgCl_2_, 1.2 mM KH_2_PO_4_, 1 mM CaCl_2_, 10 mM glucose, pH 7.4, at 37 °C) supplemented with 0.006% pluronic acid. Fura-2-loaded cells were placed in a continuously perfused recording chamber mounted on the stage of an epifluorescence microscope (Nikon Diaphot). Calcium measurements were then performed as previously described^30^.

### Extracellular lactate measurement

Cell supernatants were collected and directly frozen. Quantification of L-lactate levels was based on two enzymatic reactions. Lactate dehydrogenase (LDH; Roche, Meylan, France) catalyzed the NAD+-mediated oxidation of lactate into pyruvate. Glutamate-pyruvate transaminase (GPT; Roche, Meylan, France) was then used to shift first reaction equilibrium by transforming the entire pyruvate into alanine and α-ketoglutarate. The amount of formed NADH was related to the quantity of lactate processed by these reactions. Briefly, 20 µL of each sample were added to 200 µL of reaction buffer (620 mM sodium carbonate, 78.7 mM L-glutamate, 0.92 mM NAD, 2 µg GPT and 2 µg LDH). The standard range was performed using lithium lactate (Sigma Aldrich). The 96 multiwell plates were then incubated at 37 °C for 30 minutes before quantifying extracellular lactate production by monitoring the increase in absorbance of NADH at 355 nm on a spectrophotometer (SPECTROstar nano, BMG LABTECH, France).

### Transfection and RNA interference (siRNA)

ON-TARGETplus Rat IF1 siRNA SMARTpool (si IF1), and ON-TARGETplus Non-Targeting Pool siRNA negative control (si CTL) were purchased from GE Dharmacon. Basic Small interfering RNA (siRNA) resuspension was performed according to manufacturer’s recommendations. Transfections of siRNA were performed in 60 mm dishes on 60% confluent F258 cells, in the presence of TransFectin Lipid Reagent (BioRad). Per dish, siRNA (100 nM) and 12.5 µl TransFectin lipid reagent were applied in a final volume of 2.5 ml Opti-MEM. Six hours later, the medium was renewed with the normal medium. Cells were then passaged in order to be treated during exponential phase, as described above.

### Glucose oxidation assay

Glucose oxidation levels were monitored as previously described^22^.

### Cell toxicity estimation

Cell toxicity was measured by analysis of chromatin condensation and fragmentation in the cell nucleus as previously described^21^.

### Statistical analysis

All data were obtained from a minimum of three independent experiments. They were quoted as mean ± SD. Analysis of variance followed by Newman–Keuls test was used to test the effects of B[a]P. Differences were considered significant at the level of *P* < 0.05. All statistical analyses were performed using GraphPad Prism 5.01 Software (GraphPad Software, San Diego, USA).

## Results

### High IF1 expression is correlated with liver cancer progression and gene expression related to glycolytic metabolism in human hepatocarcinoma

**Figure 1:**
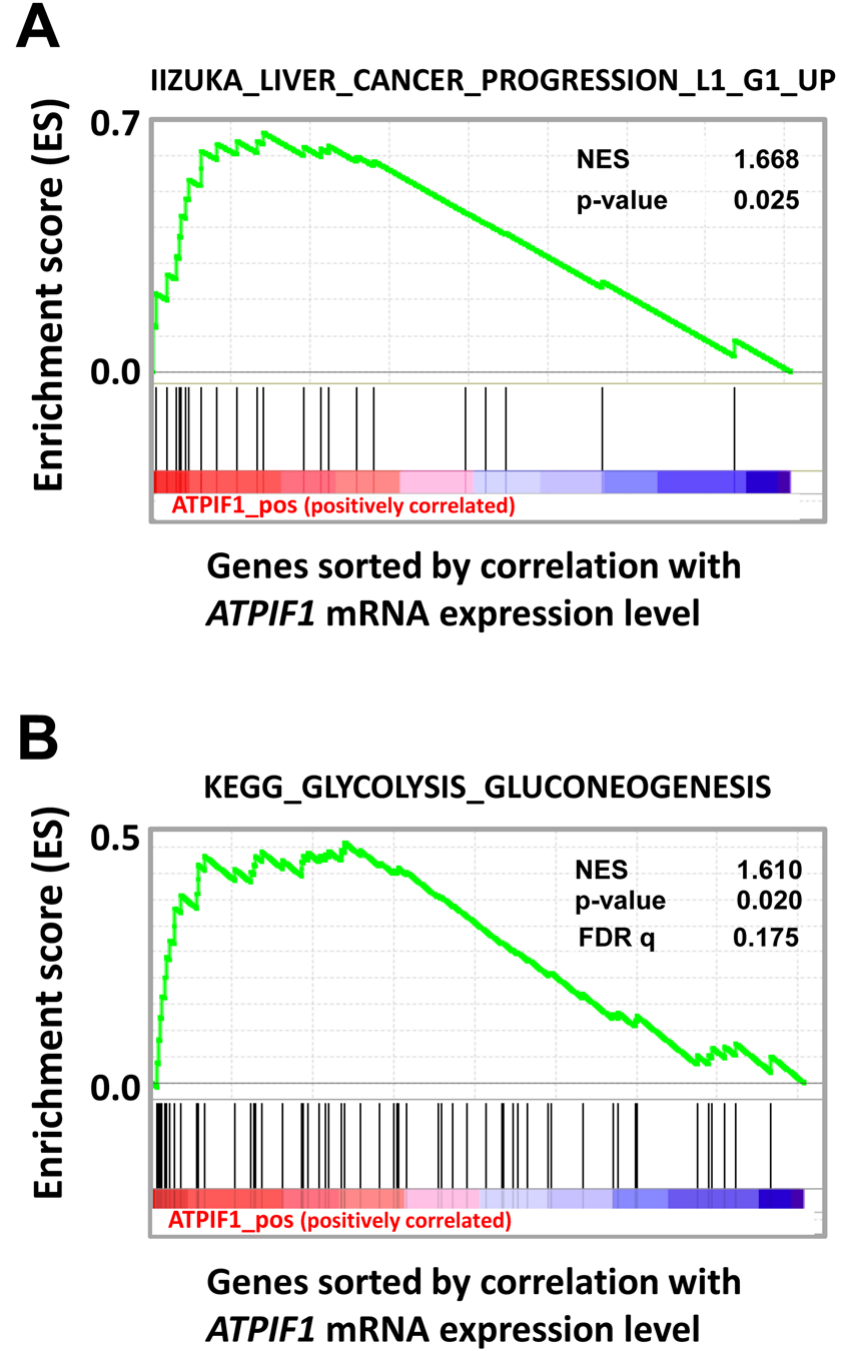
GSEA profiles showing a significant enrichment of gene sets associated with liver cancer progression (**A**), and glycolysis (KEGG, (**B**)) in human HCC with high levels of *ATPIF1* mRNA (GSE20238). See heat-map in Supplementary Fig. S1.

We first explored by GSEA the potential contribution of *ATPIF1* expression in human liver cancer progression (91 human HCV related-hepatocarcinoma, GSE20238, as previously described^25^). As shown in Fig. 1A, data mining revealed a positive correlation between high IF1 mRNA expression and the progression of non-tumor (L1) to well differentiated liver tumor (G1) (with a normalized enrichment score of 1.668, and a p value of 0.025). Consistent with the reported role of ATPIF1 in HCC metabolism *in vitro* ^14^, further GSEA analysis of GSE20238 pointed to a positive correlation between high IF1 expression and high glycolytic metabolism in human HCC (p value of 0.02 and normalized enrichment score = 1.61; Fig. 1B and Supplementary Fig. S1). The false-discovery rate (FDR) q-value of 0.175 is considered significant^24^. Altogether, these studies indicated that a high expression of IF1 was associated with both liver cancer progression and high glycolysis in human.

### *In vivo* effect of the 16 US-EPA PAH mixture and *in vitro* effect of B[a]P on hepatic IF1 expression

**Figure 2:**
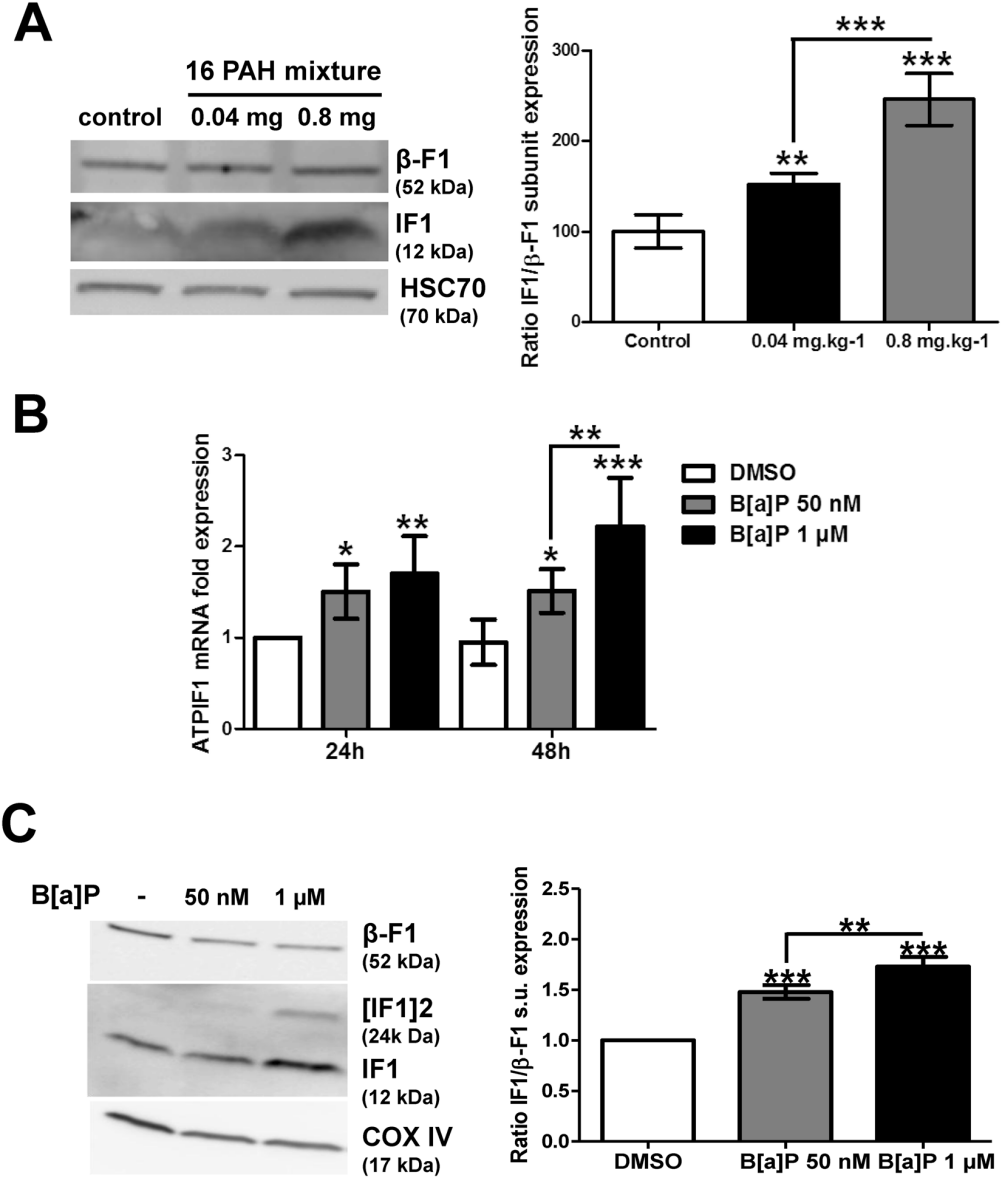
Effects of PAHs on IF1 expression both *in vivo* and *in vitro*. (**A**) The levels of IF1 protein and of the β-F1 subunit of the F0F1ATPase were analyzed by western blotting in liver tissues from rats exposed to 0.04 or 0.8 mg.kg^−1^ of a 16 PAH mixture. HSC70 was used as loading control. (**B**) IF1 mRNA expression was monitored in F258 cells treated with B[a]P (50 nM or 1 µM) for 24 or 48 hours. (**C**) The IF1 protein content of mitochondrial fractions of B[a]P-exposed F258 cells was evaluated by western blotting. Histogram gives the results of densitometric analysis of IF1 protein level relative to β-F1 subunit protein level. COXIV was used as loading control. Number of independent experiments = 3 for all conditions. *p < 0.05, **p < 0.01, ***p < 0.001, DMSO *vs* B[a]P-treated cells, unless otherwise quoted.

Based upon the relevance of IF1 in liver tumor progression, we decided to test the *in vivo* effect of a mixture of the 16 PAHs listed as “priority” compounds by the US-EPA, because of their occurrence in the environment and their potential toxicity. Thus, the hepatic IF1 protein level was evaluated in liver tissue samples collected from rats exposed through diet for 90 days at two doses (0.04 mg/kg and 0.8 mg/kg), and compared to untreated animals. Interestingly, an increase in IF1 level was detected with a dose-dependent effect (Fig. 2A). As B[a]P is the prototype of these PAHs and as it is a well-known carcinogen for human^20^, the effect of this molecule was tested on IF1 mRNA expression in rat liver epithelial F258 cells. Indeed, B[a]P was recently found to induce a metabolic reprogramming related to survival signal notably in these cells^22^. After a treatment of 24 and 48 h with two concentrations of B[a]P, a significant induction of IF1 mRNA expression was observed as soon as 24 h; an even stronger effect was detected at 48 h with B[a]P 1 µM (Fig. 2B). Regarding protein level, IF1 was detected mainly in the mitochondrial fraction compared to the cytosolic fraction in F258 cells (Supplementary Fig. S2A). Cell exposure to both concentrations of B[a]P for 48 h resulted in a dose-dependent increase in the mitochondrial IF1 protein level, as confirmed by the densitometric analysis of the ratio between IF1 and its target β-F1 subunit (Fig. 2C). Interestingly, a band corresponding to IF1 dimers was also detected, with a strong intensity at 1 µM, suggesting an increased proportion of the active form of IF1 upon B[a]P exposure. A dose-dependent effect of B[a]P (48 h) on IF1 protein level was also detected in total lysates from Hepa1c1c7 cells (Supplementary Fig. S3A), a mouse liver hepatoma cell line in which B[a]P was also found to increase glycolysis^22^. It is also noteworthy that another carcinogenic PAH, the 7,12-Dimethylbenz(a)anthracene (DMBA), also elicited an increase in IF1 protein level in F258 cells (Supplementary Fig. S3B). Altogether, these results clearly pointed to IF1 as a new target of PAHs, in particular of B[a]P, in liver.

### B[a]P metabolism would not be responsible for up-regulation of IF1 protein level

**Figure 3:**
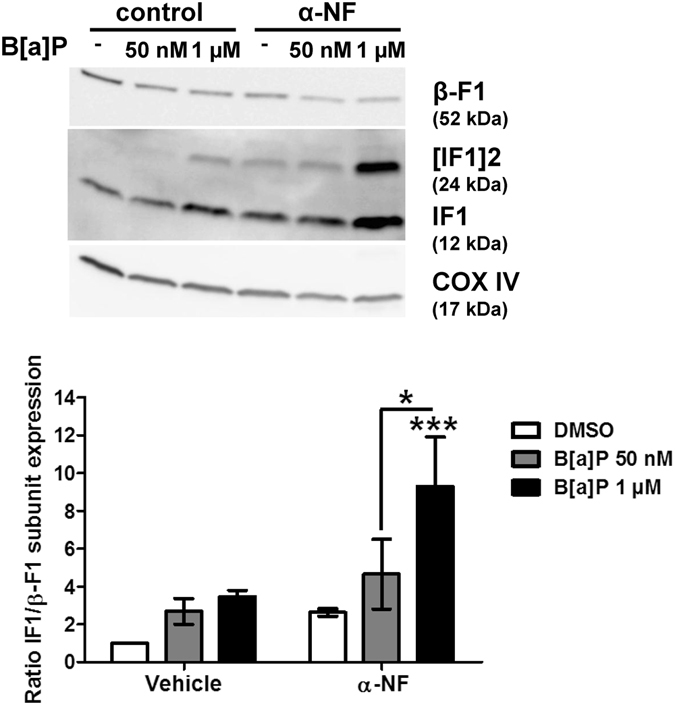
B[a]P metabolism was not involved in IF1 up-regulation in response to B[a]P. F258 cells were pre-treated for 1 hour with α-NF (10 µM) prior to co-exposure to B[a]P for 48 hours. The involvement of B[a]P metabolism in IF1 up-regulation was assessed by analyzing IF1 protein level on mitochondrial fractions by western blotting. Histogram gives the results of densitometric analysis of IF1 protein level relative to β-F1 subunit protein level. COXIV was used as loading control. Results were representative of 3 independent experiments. *p < 0.05, ***p < 0.001, DMSO *vs* B[a]P-treated cells, unless otherwise quoted.

Having demonstrated that IF1 was a new molecular target for B[a]P, we next sought to identify the origin of IF1 up-regulation. We previously demonstrated that mitochondrial hyperpolarization as well as glycolytic shift induced by B[a]P in F258 cells were dependent upon B[a]P metabolism^22, 31^. We thus tested the effect of α-naphthoflavone (α-NF; 10 µM), an inhibitor of B[a]P metabolism on the mitochondrial IF1 protein level. α-NF did not prevent IF1 up-regulation but rather enhanced it (Fig. 3). As α-NF is known to inhibit both the cytochromes P450 involved in B[a]P metabolism as well as AhR (a cytosolic receptor known to be activated by B[a]P), CH223191 (10 µM; to inhibit AhR) was tested; as illustrated in Supplementary Fig. S2A, it did not prevent the B[a]P-increased mitochondrial IF1 protein level. In contrast, it prevented the B[a]P-triggered induction of IF1 mRNA expression (Supplementary Fig. S2B). Moreover, TCDD which is a strong activator of AhR, was ineffective towards IF1 protein level when used alone (Supplementary Fig. S2C). In total, these results showed that B[a]P enhanced the mitochondrial IF1 protein level by a pathway that would involve neither B[a]P metabolism, nor AhR activation.

### The β2-adrenergic pathway would be involved in IF1 up-regulation upon B[a]P exposure

**Figure 4:**
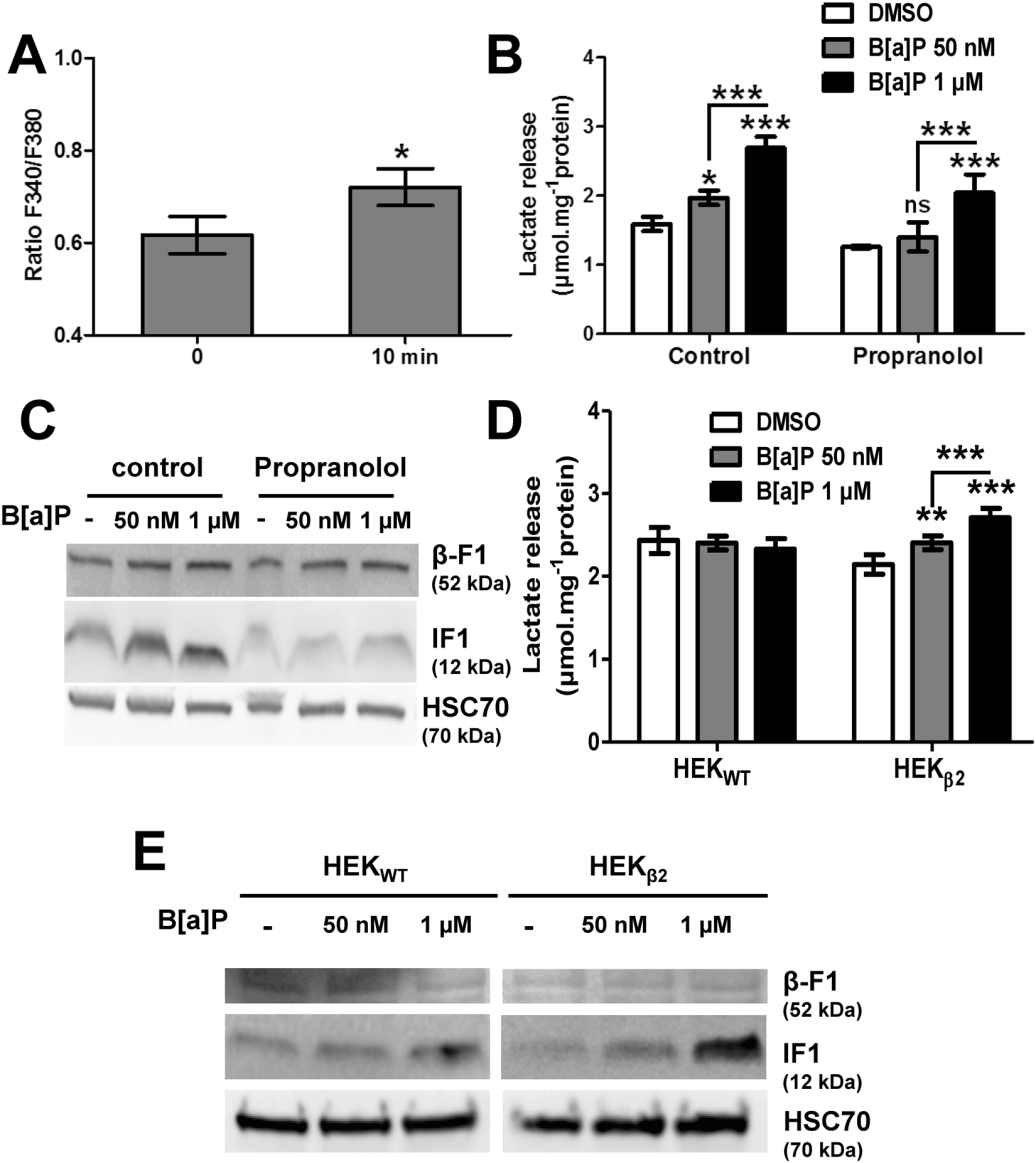
B[a]P-induced IF1 induction could occur through β2-adrenergic pathway stimulation. (**A**) Effects of B[a]P (50 nM) on calcium was assessed by microspectrofluorimetry after staining F258 cells with the Fura-2-AM probe. The ratio F340/F380 reflects intracellular Ca^2+^ concentration. *p < 0.05, DMSO vs B[a]P-treated cells. (**B**,**C**) F258 cells were pre-treated or not with the β-receptor inhibitor propranolol (10 µM) for 1 hour prior to co-exposure to B[a]P for 48 hours. The B[a]P-induced glycolytic shift (**B**) was investigated by monitoring extracellular lactate, and the IF1 protein level (**C**) was analyzed on total lysates by western blotting. A role for β2-adrenergic receptor in the B[a]P (50 nM or 1 µM, 48 h)-induced extracellular lactate (**D**) or total IF1 protein level (**E**), was evaluated in both HEK_WT_ cells (not expressing β2-adrenergic receptor), and HEK_β2_ (in which the receptor is overexpressed). HSC70 was used as loading control. Number of independent experiments = 3 for all conditions. *p < 0.05, **p < 0.01, ***p < 0.001, DMSO vs B[a]P-treated cells, unless otherwise quoted.

The next set of experiments was performed in order to test the possible involvement of the β2-adrenergic pathway in the regulation of the protein level of IF1 by B[a]P. As an increase in intracellular calcium concentration has been previously observed upon B[a]P-induced β2ADR activation^32^, the effect of B[a]P (50 nM) on calcium concentration was first assayed under our experimental conditions, by using Fura-2-AM as calcium sensitive fluoroprobe, and microspectrofluorimetry. As shown in Fig. 4A, an increase in calcium was triggered by B[a]P in F258 cells following 10 min of exposure. This then led us to test the possible involvement of β2ADR in the B[a]P effects on IF1 protein level; propranolol, a non-selective β blocker, was thus used to inhibit this pathway. We first showed that a pre-treatment (1 h) with propranolol (10 µM) followed by co-treatment with B[a]P (48 h), prevented not only the release of lactate, especially at the lowest B[a]P concentration (Fig. 4B), but also the increase in IF1 protein level (Fig. 4C). The use of a selective β2-blocker, ICI-118,551, gave similar results on IF1 level (n = 2; data not shown). Finally, the effect of B[a]P was assayed in HEK293 cells overexpressing β2ADR. Indeed, these cells are known to express a very low basal level of β2ADR^33^; they were then transfected either with control plasmid (HEKwt) or with a β2ADR plasmid (HEKβ2), as previously described^32^. As shown in Fig. 4D, B[a]P was responsible for a dose-dependent release of lactate from HEKβ2 cells, with no effect in HEKwt. When looking at the IF1 protein level in these two cell lines, the effect of B[a]P on IF1 was more marked in HEKβ2 compared to HEKwt cells (Fig. 4E). Altogether, these results strongly suggested a role for β2ADR in the regulation of IF1 protein level by B[a]P.

### IF1 exhibits a pivotal role in B[a]P propensity to induce cell survival

**Figure 5:**
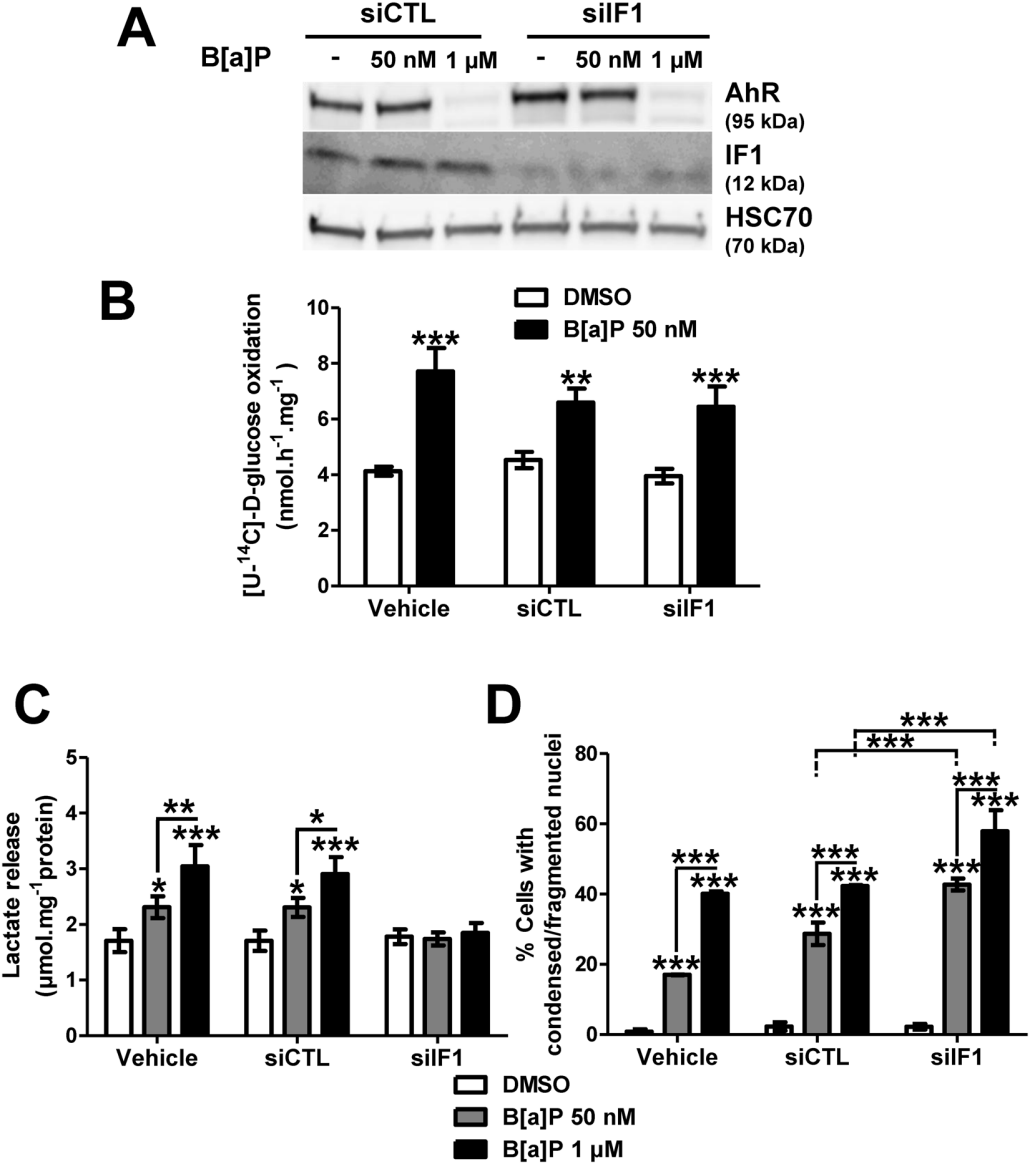
IF1 upregulation following B[a]P exposure acts to promote F258 cell survival. (**A**) Western blotting analysis was performed to control siIF1 inhibitory efficacy on IF1 protein level expression compared to the Non Targeting siRNA (siCTL). AhR expression was analyzed to ensure the specificity of Si targeting. HSC70 was used as loading control. (**B**) Impact of IF1 silencing on glucose oxidation was quantified by measuring transfer of ^14^C radioactivity from glucose to CO_2_. Cells were treated with B[a]P (50 nM) for 48 h. (**C**) Role for IF1 in the B[a]P-induced glycolytic shift was evaluated by measuring extracellular lactate upon IF1 silencing by siRNA. (**D**) B[a]P toxicity was evaluated in IF1 silenced cells by counting cells with fragmented or condensed chromatin following Hoechst 33342 staining. Number of independent experiments = 3 for all conditions. *p < 0.05, **p < 0.01, ***p < 0.001, DMSO *vs* B[a]P-treated cells, unless otherwise quoted.

An increase in IF1 protein level has been previously related to Warburg effect and cell survival^15^. In order to test whether the B[a]P-increased mitochondrial IF1 protein level was involved in both the glycolytic shift and cell survival signaling we recently reported^21, 22^, we decided to test the role of IF1 towards both glycolytic shift and cell death by using a siRNA approach. First, western blotting experiments were carried out in order to validate the siRNA targeting IF1 (siIF1) used. As shown in Fig. 5A, transfecting cells with siIF1 markedly reduced the expression level of this protein, compared to conditions with control siRNA (siCTL). Glucose oxidation, previously shown to be enhanced by B[a]P^22^, was then evaluated in siIF1-or siCTL-transfected cells. Data from Fig. 5B indicated that the B[a]P (50 nM, 48 h)-increased glucose oxidation did not rely on IF1. In contrast, silencing IF1 fully prevented the increase in lactate release induced by B[a]P (48 h), whatever the concentration used (Fig. 5C). Finally, analysis of cell death by cell nucleus staining with Hoechst 33342 showed a significant increase in B[a]P-induced cell death when cells were transfected with siIF1 (Fig. 5D). Such an increase did not seem to rely upon an over-activation of the p53 pathway (Supplementary Fig. S4). Altogether, these results pointed to a crucial role for IF1 in B[a]P-induced glycolytic shift and cell survival.

## Discussion

High overexpression of IF1 has been reported in numerous human cancers, such as gastric cancer^34^, colon, lung, breast and ovarian carcinomas^13^, as well as hepatocarcinoma^14^, which makes it as an important cancer biomarker. Regarding its role in carcinogenesis, such a high expression has been clearly evidenced as notably inducing metabolic reprogramming towards glycolysis and cell survival^11, 15, 35^. However, as stressed by Sánchez-Arago *et al*.^13^, the question that still remains to be fully solved is how IF1 is upregulated in cancer. This is in this context that our study was performed in order to evaluate the possible impact of known environmental carcinogens, namely PAHs. The present data clearly evidenced that rat exposure (90 days) to a low dose of the 16 US-EPA PAH mixture, which is known to have deleterious effects, was capable of increasing *in vivo* the IF1 protein level in liver, as shown by the marked increase in the monomeric 12 kDa form. Furthermore, we found that the PAH prototype B[a]P, which is a well-recognized human carcinogen^20^, up-regulated IF1 level *in vitro* in both F258 and Hepa1c1c7 cells. Note that 7,12-Dimethylbenz(a)anthracene, another known carcinogenic PAH, also upregulated IF1 in F258 cells. In this context, our study is the first one to identify environmental carcinogens as exogenous regulators of IF1 level.

Whereas some intracellular regulators of IF1 mRNA expression have been reported, such as the transcription factor NFκB notably in hepatocarcinoma^14^, or the nuclear receptor pregnane X receptor (PXR) in the liver from rats treated by the synthetic steroid pregnenolone 16α-carbonitrile^36^, little information is available regarding the involvement of other endogenous transcriptional regulators. HIF-1α would control IF1 protein level in Clone 9 cells, a non-transformed rat hepatic epithelial cell line, upon hypoxia; however, whereas a decrease in IF1 mRNA expression was paralleled by a decrease in HIF-1α mRNA expression in rat liver during sepsis, direct evidence for the transcriptional regulation of ATPIF1 gene by this transcription factor was not sought^37^. Our present study would point to AhR as possibly involved in the transcriptional regulation of *ATPIF1* gene. Indeed, by using the specific AhR inhibitor CH223191, induction of IF1 mRNA expression by B[a]P was found to be fully inhibited. However, this possible transcriptional regulation would not play any role in the increase of mitochondrial IF1 protein level induced by B[a]P exposure, since inhibiting AhR was ineffective on this increase. In line with this result, AhR activation by TCDD alone had no effect under control conditions (Supplementary Fig. S2C). Our previous work indicated that B[a]P metabolism by CYP1B1 could play a role in metabolism reprogramming^21, 22^. However, as for CH223191, α-naphthoflavone did not prevent IF1 increase. In this context, B[a]P metabolism would not be involved, even though this will need to be firmly validated.

**Figure 6:**
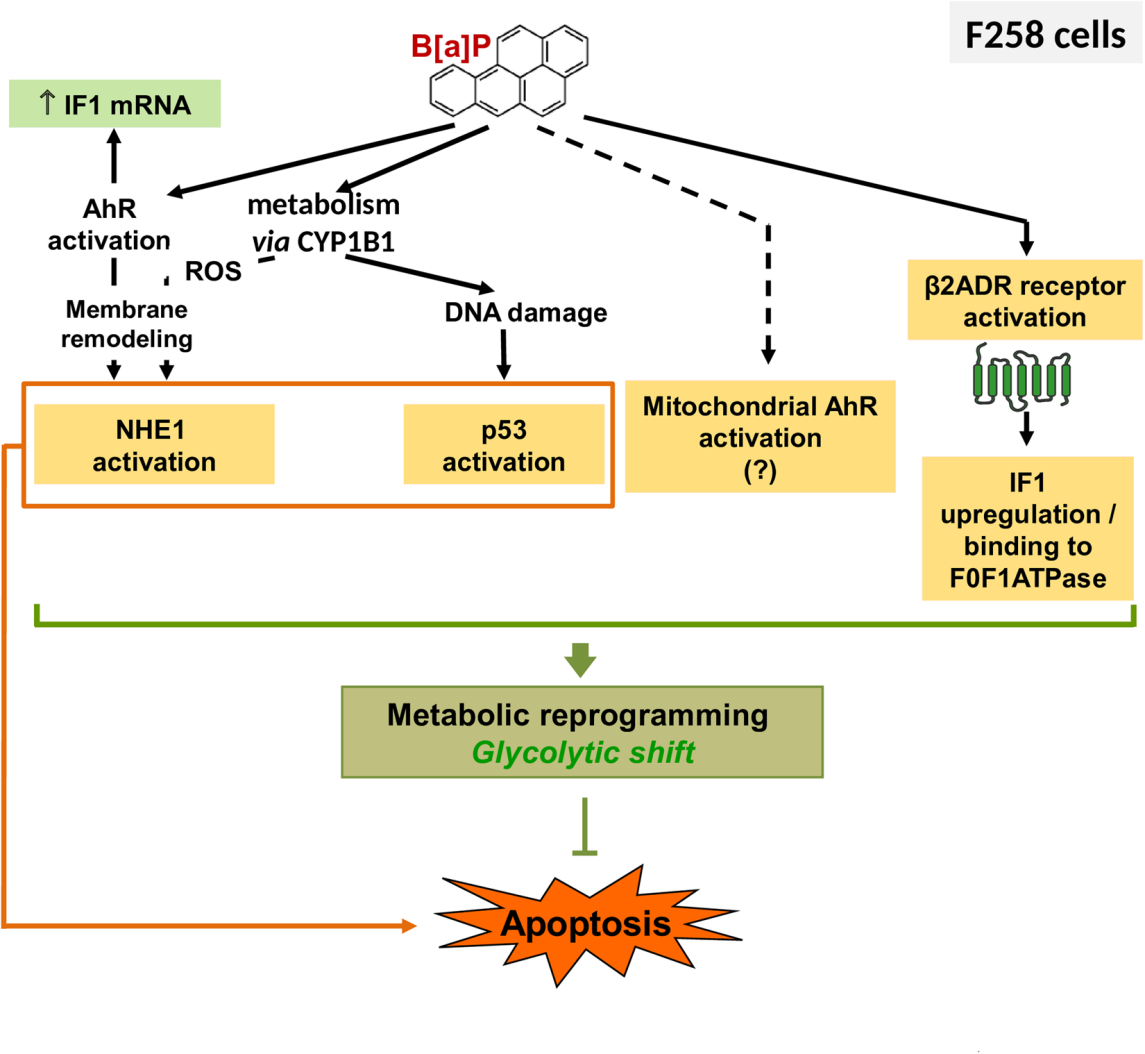
A proposed model for the B[a]P-mediated metabolic reprogramming and its role in cell fate determination in F258 rat hepatic epithelial cells. B[a]P metabolism, *via* the constitutively expressed CYP1B1, leads to the activation of NHE1 transporter and p53 pathway, both triggering apoptotic cell death. Note that AhR activation by B[a]P, along with the reactive oxygen species (ROS) production related to CYP metabolism, are involved in NHE1 activation *via* membrane remodeling^49^. Beside promoting apoptosis, activation of NHE1 and p53 also appeared to be involved in the B[a]P-elicited metabolic reprogramming^22^, thus inducing cell survival. B[a]P would also induce a β2-adrenergic receptor-dependent activation of IF1, the physiological inhibitor of the F1F0ATPase, which appears to be involved in the related glycolytic shift and cell survival. Activation of AhR by B[a]P might be involved in the upregulation of IF1 mRNA level, but without any incidence on the mitochondrial IF1 protein level. Along with its classical and well described transcription factor activity, a mitochondrial fraction of AhR might also participate to the overall metabolic reprogramming.

Direct activation of β2ADR by B[a]P has been previously described, thus leading to a rapid increase in intracellular calcium concentration^32^; moreover, this increase in intracellular calcium was found to be AhR-independent^30^. Besides, β2ADR is known to control mitochondria biogenesis and function^38, 39^. Regarding this latter aspect, it is worth emphasizing that β2ADR was reported to afford cardioprotection by interfering with mitochondrial dysfunction upon oxidative stress induced by doxorubicin^38^. In this context, regulation by β2ADR of the B[a]P-elicited increase in IF1 protein level was tested by using chemical inhibitors in F258 cells or by using β2ADR-expressing HEK293 cells. From our results, it appeared that early activation of the β2ADR pathway by B[a]P might be involved in the control of mitochondrial IF1 protein level (Fig. 6). Regarding that point, it is noteworthy that a recent study has revealed a role for the β-adrenergic receptor/cAMP/PKA pathway in controlling the phosphorylation state of IF1, thereby preventing its binding to the F0F1-ATPase^40^.

IF1 activation has been previously related to a drop in mitochondrial matrix pH, thus allowing the appearance of a dimeric form, which then binds to the βF1 subunit of the F1F0ATPase, thereby inhibiting its hydrolase activity^8^. As B[a]P was reported to trigger a mitochondrial matrix acidification, from approximately 8 to 6.5, in F258 cells^22^, this could explain the appearance of a 24 kDa band presently detected after electrophoresis, thus revealing the presence of active IF1 dimers. Despite the IF1 up-regulation and dimer appearance, only a trend towards decrease for complex V activity was found (decrease by about 12%; data not shown). However, it would be worth measuring specifically its hydrolase activity upon B[a]P exposure; indeed, a decrease in this activity of complex V is known to afford some cell protection towards cell death by limiting ATP consumption^41^. Previous studies have revealed that IF1 overexpression leads to Warburg effect, a well-known hallmark of cancer^11, 15^. Besides, our recent work has shown that B[a]P triggers a Warburg effect involved in cell survival in F258 cells^22^. Here we further show that silencing IF1 blocks the B[a]P-induced enhanced extracellular lactate release, and would thus been involved in the B[a]P-induced glycolytic shift. However, the boost in glucose oxidation elicited by B[a]P^22^ does not seem to rely on IF1. As we have previously shown that (i)-NHE1 controls both lactate release and glucose oxidation induced by B[a]P, and that (ii)-AhR might also be involved^22^, it then appears that the overall metabolic reprogramming induced by B[a]P might be more complex than first believed (Fig. 6), and would thus deserve further investigation. Besides being involved in the B[a]P-induced glycolytic reprogramming, IF1 might also exert its pro-survival effects through regulating the mitochondrial permeability transition pore PTP. Indeed, Antoniel *et al*.^42^ recently pointed out some striking similarities between inhibition of the permeability transition pore (PTP) opening and IF1 regulation, both known for example to rely on mitochondrial matrix pH. Indeed, matrix acidification has been shown to prevent the PTP opening^43^. In support to such a possible effect of IF1 on PTP, it is worth stressing that the B[a]P-induced apoptosis was found to occur without any cytochrome c release in F258 cells^23^. Nevertheless, IF1 effects are still controversial and seem to be highly dependent on the biological context. In particular, Fujikawa *et al*.^44^, and more recently Barbato *et al*.^45^ rather demonstrate a role for IF1 overexpression in mitochondrial membrane depolarization related to an enhancement of OXPHOS rate. Finally, overexpression of IF1 is also known to greatly impact on mitochondria network dynamics^16, 17, 44, 46^. Hyperfused mitochondria, whose formation depends on maintenance of Δψm, can transiently buffer the effects of respiratory chain dysfunction; such mitochondria would not be processed by mitophagy, thereby promoting a selective rescue mechanism^47, 48^. Regarding that point, mitotracker Red staining (Supplementary Fig. S5A) and TEM (Supplementary Fig. S5B), evidenced elongated mitochondria in B[a]P-treated F258 cells. In this context, one might also suggest a role for IF1 in this enhanced mitochondrial fusion process, modeled by a marked mitochondrial elongation, as previously reported^16, 17^.

In conclusion, although B[a]P is known to be involved in the different phases of tumor development, contribution of B[a]P-induced mitochondrial dysfunction to cell transformation, and in the following onset of oncogenesis still needs to be clarified. Here we show that IF1, the physiological inhibitor of the F0F1ATPase is a new target for PAHs, even at low concentrations, and that PAH-regulated IF1 is linked to glycolytic shift and survival. Interestingly, our datamining analysis from transcriptomics data issued from human hepatocarcinoma clearly reveals positive correlations between high IF1 expression on one hand, and glycolysis and tumor progression on the other hand. In this context, IF1 upregulation might then be a new means for PAHs to drive carcinogenesis. We therefore suggest that IF1 might be a suitable marker for PAH-induced carcinogenesis.

## Electronic supplementary material

Supplementary information
