# Supplementary material for: Role for the ATPase inhibitory factor 1 in the environmental carcinogen-induced Warburg phenotype
Source: Sci Rep. 2017 Mar 15;7:195. doi: 10.1038/s41598-017-00269-7 (PMC5428028; doi:10.1038/s41598-017-00269-7)
Supplement: Supplementary file 1 — Supplementary information [file 41598_2017_269_MOESM1_ESM.pdf]

SUPPLEMENTARY INFORMATION

Role for the ATPase inhibitory factor 1 in the environmental carcinogen-induced Warburg phenotype

Kévin Hardonnière[1,2], Morgane Fernier[1,2], Isabelle Gallais[1,2], Baharia Mograbi[3], Normand Podechard[1,2], Eric Le Ferrec[1,2], Nathalie Grova[4], Brice Appenzeller[4], Agnès Burel[2], Martine Chevanne[1,2], Odile Sergent[1,2], Laurence Huc[5], Sylvie Bortoli[6], Dominique Lagadic-Gossmann[1,2]

SUPPLEMENTARY METHODS

Western Blot immunoassays

For whole-cell lysates, cells were harvested and lysed for 20 min on ice in RIPA buffer supplemented with 1 mM phenylmethylsulfonyl fluoride, 0.5 mM dithiothreitol, 1 mM orthovanadate, and a cocktail of protein inhibitors (Roche). Cells were then centrifuged at 13,000*g* for 15 min at 4 °C. The resulting supernatants were collected and frozen at −80 °C or used immediately. Ten to 30 µg of whole-cell lysates were heated for 5 min at 100 °C, loaded in a 4% stacking gel, and then separated by 10% sodium dodecyl sulfate–polymerase gel electrophoresis (SDS–PAGE). Gels were electroblotted overnight onto nitrocellulose membranes (Millipore).

For mitochondrial lysates, 30 µg were heated at 100°C for 5 min and then loaded on Any kD mini format precast gels (Biorad). After migration process, gels were then electroblotted on PVDF membrane (Biorad) using the Trans-Blot Transfer System (Biorad). After membrane blocking with a Tris-buffered saline (TBS) solution supplemented with 5% bovine serum albumin, membranes were then hybridized with primary antibodies overnight at 4 °C and next incubated with appropriate horseradish peroxidase-conjugated secondary antibodies for 1 hour. Immunolabeled proteins were visualized by chemiluminescence using the LAS-3000 analyzer (Fujifilm). Image processing was performed using Multi Gauge software (Fujifilm).

Cell culture

The mouse hepatoma cell line Hepa1c1c7 (purchased from the European Collection of Cell Culture) was maintained in MEMα medium with l-glutamine without ribonucleosides and deoxyribonucleosides (Gibco, Cergy Pontoise, France), and supplemented with 10% fetal calf serum and 0.1 mg/ml gentamycin, as previously described (Holme et al., 2007; Podechard et al., 2011). Cells were seeded near confluence (90 × 103 cells/cm^2) a day before treatment and the medium changed before exposures. When using inhibitors, these were added for 1 h before B[a]P treatment, for the indicated time points.

Mitochondria network visualization using fluorescence microscopy

F258 cells, grown on coverslips, were stained with 10 nM Mitotracker Red (a mitochondria selective dye; Molecular Probes, Invitrogen Life Technologies) during the last 30 min of B[a]P exposure. Cells were then washed with PBS and fixed in 4% paraformaldehyde (Sigma-Aldrich) for 20 min and then washed with PBS. Samples were digitized with 63x or 40x fluorescence objectives (Zeiss) on the IX83 inverted microscope (Olympus; Rungis, France) equipped with an ultra-high-speed wavelenght switching system Lambda DG4 (Sutter Instrument; Novato, USA) and an ORCA Flash 4.0 CMOS camera (Hamamatsu; Massy, France) using cellSense Dimension software (Olympus).

Analysis of the mitochondria network ultrastructure using Transmission electron microscopy

After drug exposure, cells were rinsed with 0.15 M Na cacodylate buffer and fixed by drop-wise addition of glutaraldehyde (2.5%) for 1 h. After fixation, the specimens were rinsed

several times with 0.15 M Na cacodylate buffer and postfixed with 1.5% osmium tetroxide for 1 h. After further rinsing with cacodylate buffer, the samples were dehydrated through a series of graded ethanol from 70 to 100%. The specimens were infiltrated in a mixture of acetone–Eponate (50/50) for 3 h and then in pure Eponate for 16 h. Finally, the specimens were embedded in DMP30–Eponate for 24 h at 60 °C. Sections (0.5 µm) were cut on a Leica UC7 microtome and stained with toluidine blue. Ultrathin sections (90 nm) were obtained, collected onto copper grids, and counterstained with 4% uranyl acetate and then with lead citrate. Examination was performed with a JEOL 1400 transmission electron microscope operated at 120 kV.

References

Holme JA, Gorria M, Arlt VM, Ovrebø S, Solhaug A, Tekpli X, Landvik NE, Huc L, Fardel O, Lagadic-Gossmann D. Different mechanisms involved in apoptosis following exposure to benzo[a]pyrene in F258 and Hepa1c1c7 cells. Chem Biol Interact. 2007 Apr 5;167(1):41-55.

Podechard N, Tekpli X, Catheline D, Holme JA, Rioux V, Legrand P, Rialland M, Fardel O, Lagadic-Gossmann D, Lecureur V.Mechanisms involved in lipid accumulation and apoptosis induced by 1-nitropyrene in Hepa1c1c7 cells. Toxicol Lett. 2011 Oct 30;206(3):289-99.

Supplementary Figure S1

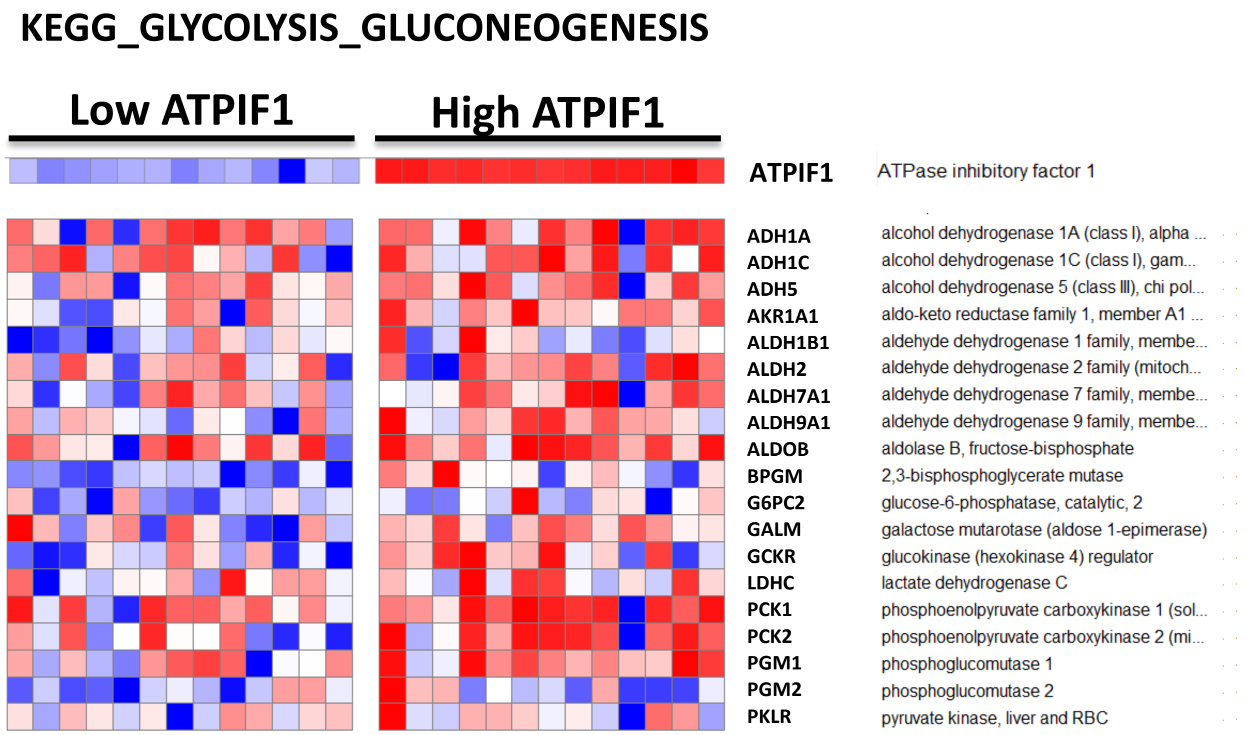

HEPATOCELLULAR CARCINOMA (91 patients)

Heat-map highlights changes in the constituent genes of the glycolysis/gluconeogenesis pathway in human HCC with high and low levels of *ATPIF1* mRNA (GSE20238).

Supplementary Figure S2

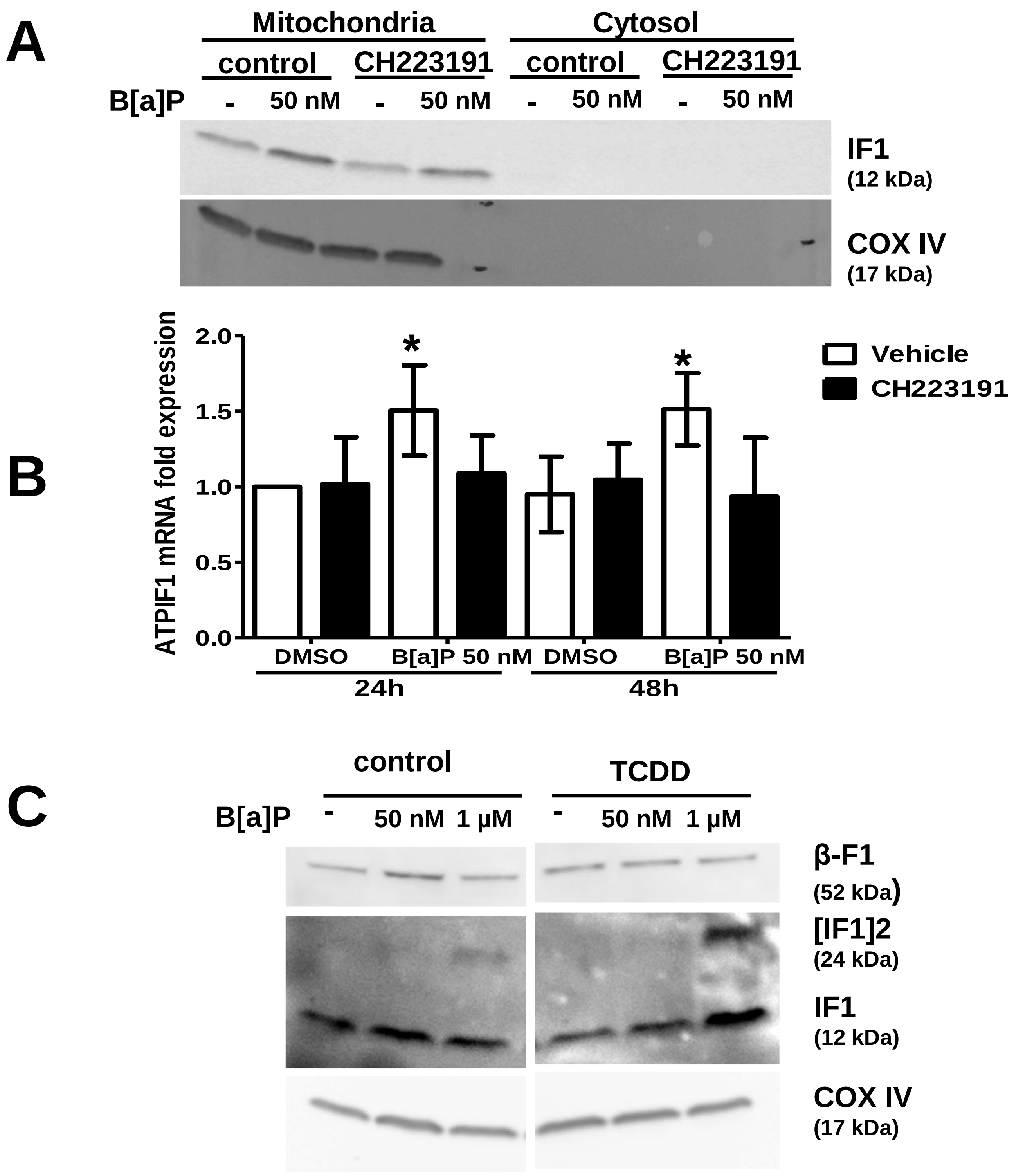

Impact of AhR inhibition (A, B) or activation (C) on IF1 protein level (A, C) and mRNA expression (B). (A) F258 cells were pre-treated for 1 hour with CH223191 (10 µM; an AhR inhibitor) prior to co-exposure to B[a]P for 48 hours. (A, C) IF1 protein level was evaluated on mitochondrial fractions by western blotting analysis. (B) Effects of CH223191 on the B[a]P-induced IF1 mRNA expression were assessed by RT-qPCR. *: $p<0.05$ B[a]P *versus* DMSO. (C) F258 cells were treated for 1 hour with TCDD (10 nM; a strong AhR ligand) prior to co-exposure to B[a]P for 48 hours. Number of independent experiments = 3 for all conditions, except for (A) (n = 2).

Supplementary Figure S3

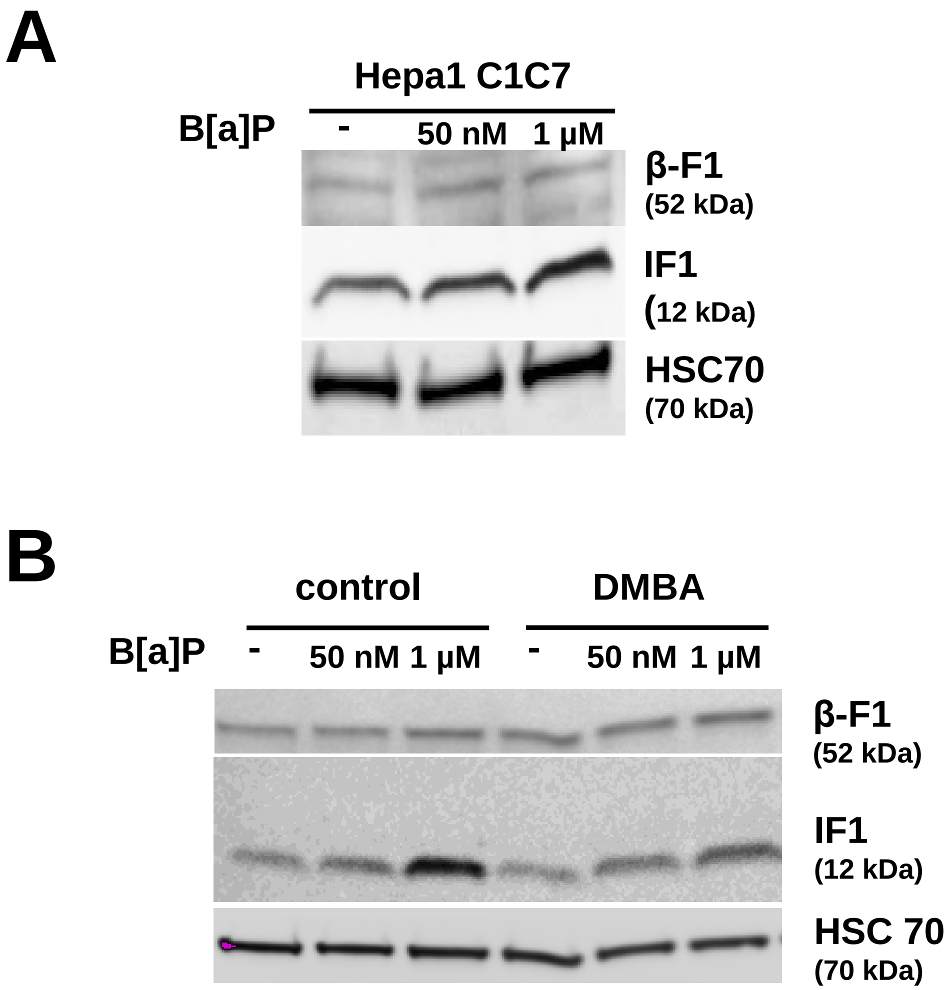

(A) Effects of a 48h-treatment with B[a]P on IF1 protein expression assessed by western blotting on total cell lysates from Hepa1c1c7 mouse hepatoma cells. (B) Effects of another carcinogenic PAH, the 7,12-Dimethylbenz(a)anthracene (DMBA), on IF1 protein expression assessed by western blotting on total cell lysates from F258 rat hepatic epithelial cells. HSC70 was used as loading control. Number of independent experiments = 3 for all conditions.

Supplementary Figure S4

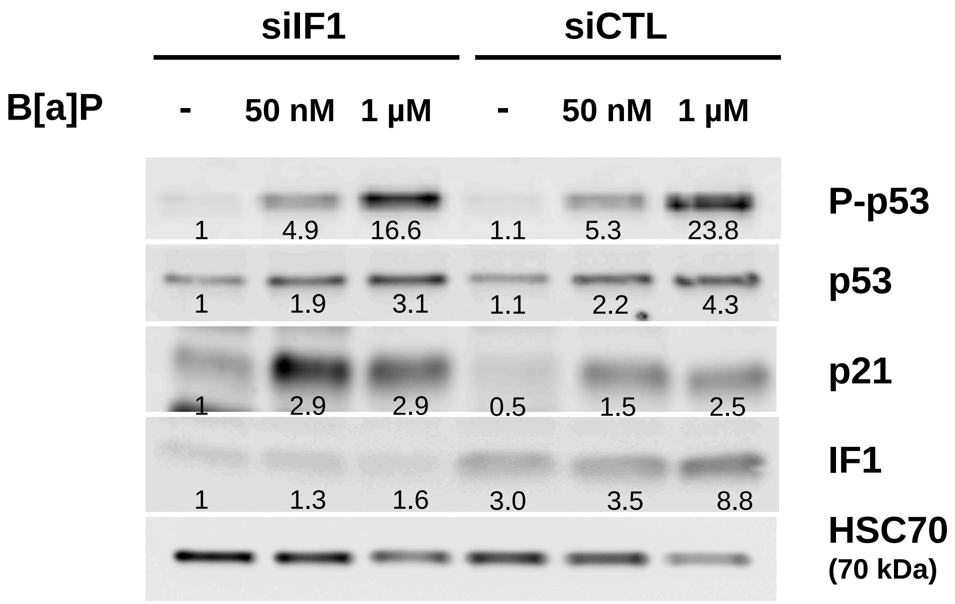

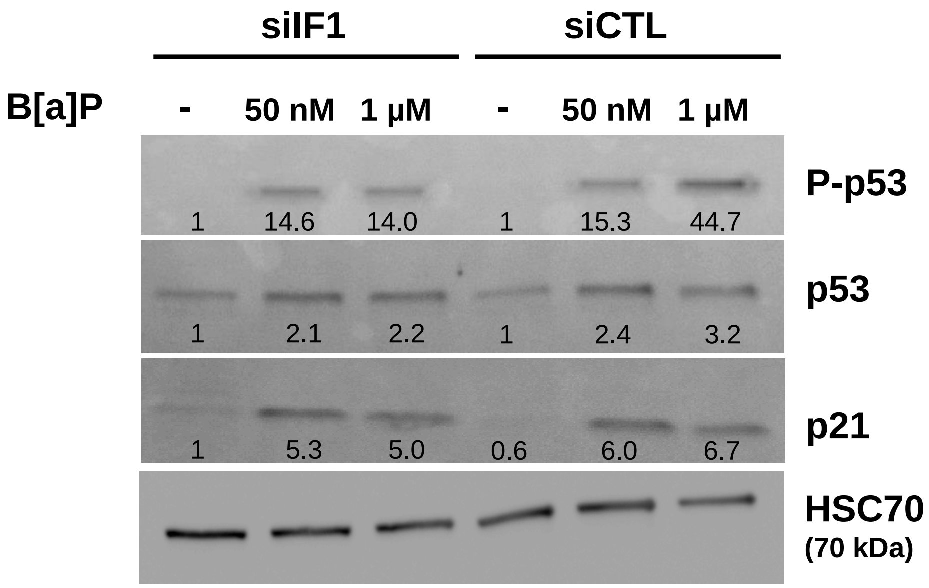

Effects of silencing IF1 gene expression by Si RNA on the B[a]P (48h)-induced p53 pathway activation. The protein levels of phospho-p53 (P-p53), total p53, p21 (a p53 target gene) and IF1 were assessed by western blotting on total cell lysates from F258 rat hepatic epithelial cells, treated either with SiRNA targeting IF1 (siIF1) or non targeting siRNA (SiCTL). HSC70 was used as loading control. The fold induction (densitometric value given relative to HSC70 level and arbitrary set to 1 for B[a]P-untreated, siIF1-treated cells) are given next to the plots. Two representative blots are shown. Number of independent experiments = 3 for all conditions.

Supplementary Figure S5

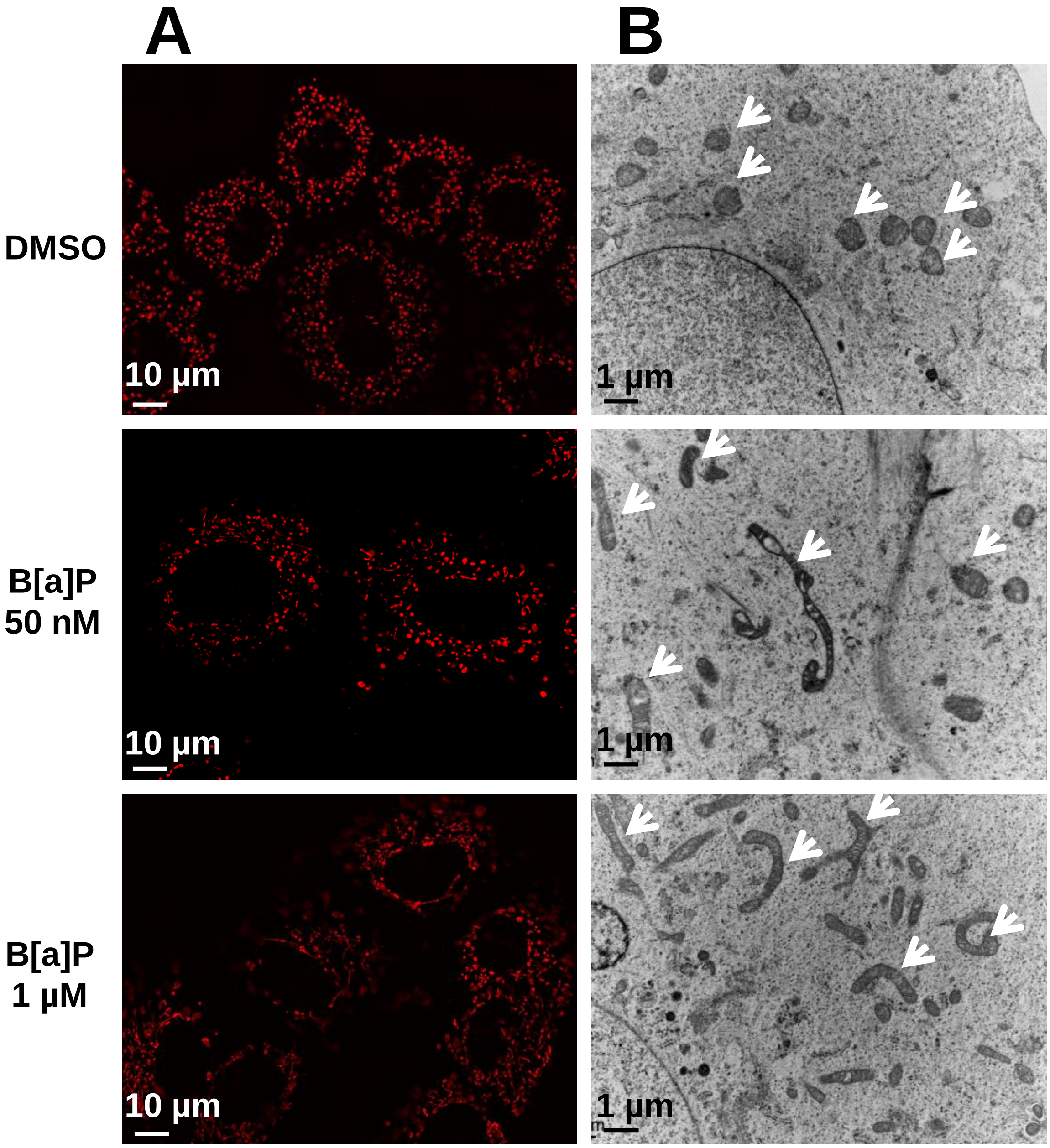

Effect of B[a]P on the mitochondrial network structure. F258 cells were treated or not with B[a]P (50 nM or 1 µM) for 48 hours before microscopy experiments. (A) Overall structure of mitochondrial networks were digitized on the IX83 inverted microscope (63X magnification) after MitoTracker Red staining (10 nM). Number of independent experiments = 3 for all conditions. (B) Ultra-thin sections (90 nm) of each culture were observed under transmission electron microscopy (TEM). Arrows indicate mitochondria. Number of independent experiments = 2 for all conditions.
